# London Medical Committee

**Published:** 1913-11-15

**Authors:** 


### LONDON MEDICAL COMMITTEE.

On Thursday last, November 6, at a meeting of the London Medical Committee (which is entirely non-panel in its constitution) it was decided to send representatives to the Conference of Non-Panel Associations which is to be held in London on Saturday, November 29. This Conference is being called by the National Medical Union, a non-panel body having its centre in Manchester, with the assistance of Non-Panel Associations in London and the provinces. The committee appointed the chairman, Dr. Fred. J. Smith, Dr. Bernard O'Connor, barrister-at-law, and the hon. secretary, Mr. Dorrell (45 Welbeck Street, W.), to be the representatives at the Conference. A report was received that two new Non-Panel Associations had been formed —one for Lambeth, and the other for Islington and St. Pancras. Also reports of meetings and of the strength of membership were received from Wandsworth, Hampstead, and Lewisham Non-Panel Associations. A letter was received from a medical man in the Wandsworth district, who has unrivalled opportunity of observing the working of the Insurance Act in the poorer districts, showing how thoroughly the insured dislike the Act and the panel system with its restricted choice of doctor, and how "hundreds of panel patients come to me" either because they will not go to a panel doctor or do not wish to repeat the experience.

The committee adjourned until Monday, November 17.

